# Risk Factors for Inadequate Bowel Preparation During Colonoscopy in Nigerian Patients

**DOI:** 10.7759/cureus.17145

**Published:** 2021-08-13

**Authors:** Emeka Ray-Offor, Nze Jebbin

**Affiliations:** 1 Digestive Disease Unit, Oak Endoscopy Centre, Port Harcourt, NGA; 2 Colorectal and Minimal Access Surgery Unit, Department of Surgery, University of Port Harcourt Teaching Hospital, Port Harcourt, NGA

**Keywords:** colonoscopy, bowel preparation, predictors, outcome, sub-sahara africa

## Abstract

Background

The past few decades have witnessed the introduction of various innovative technologies into colon study by colonoscopy. A well-prepared bowel is crucial to their effective utilization. An inadequate bowel preparation during colonoscopy is associated with increased technical difficulties, enhanced risks of perforation, longer examination durations, reduced adenoma detection rates, and additional costs related to repeated colonoscopies. There is a paucity of literature from Africa on the multiple patient factors that affect the quality of bowel preparation; hence, the need to identify patients at risk for inadequate bowel preparation to allow for more diligence in this special group.

Aim

To study the risk factors of inadequate bowel preparation for colonoscopy and identify the group of patients who need intensified preparation in a Nigerian population.

Methods

A case-control study of consecutive patients undergoing colonoscopy in an open access/referral-based multi-disciplinary endoscopy facility in Port Harcourt metropolis, Nigeria from March 2014 to November 2020. Consecutive adult patients who underwent colonoscopy with inadequate bowel preparation irrespective of the indication were retrospectively identified. Each case of inadequate bowel preparation while using a particular bowel preparation agent was matched with the next colon study with adequate bowel preparation (control) for the same agent in a 1:1 ratio. The variables collated were age, gender, literacy level, colonoscopy indication, medical history, bowel preparation agent, timing of endoscopy, and outcome. Statistical analysis was performed using Statistical Package for the Social Sciences (SPSS) version 25 (IBM Corp., Armonk, NY).

Results

There were 143 cases of inadequate bowel preparation during colonoscopy included in the study with an equal number of control (cases of adequate bowel preparation). The age of patients ranged from 24 years to 92 years. Bleeding per rectum - 122(42.7%), and screening for colorectal cancer - 67(23.4%), were the leading indications for colonoscopy in study patients. Bivariate analysis of cases and controls revealed significant difference in educational status, comorbidity of hypertension, and constipation (p < 0.01, p = 0.082, p = 0.143, respectively). In the multivariate analysis of risk factors, the odds ratio (OR) for secondary level of education and below was 2.54 (95% confidence interval CI 1.50-4.30; p = 0.001); hypertension - OR 1.64 (95% CI 0.98-2.73; p = 0.058); constipation - OR 1.27 (95% CI 0.52-3.10; p = 0.598).

Conclusion

The educational status of patients is a strong risk factor associated with inadequate bowel preparation for colonoscopy in this Nigerian population. There is a need for effective patient education especially for patients with a low literacy level.

## Introduction

Advances in technology have resulted in image enhancement and improved procedural performance in colonoscopy [1]. These advances are, however, dependent on the quality of bowel preparation during colonoscopy for their utilization. An adequate colonic examination is one that allows confidence that mass lesions ≥5 mm are detectable by the bowel preparation [2]. The adequacy of bowel preparation is crucial, hence, the need to identify patients at risk for inadequate bowel preparation to allow for more diligence in this special group. It has been reported that inadequate bowel cleansing is observed in approximately one-quarter of all colonoscopies [3]. Inadequate bowel preparation is associated with increased technical difficulties, enhanced risks of perforation, longer examination durations, and reduced adenoma detection rates [3,4]. Of significance are the additional costs related to repeated colonoscopies more so in a limited resource setting [5].

Different scales have been developed for clinical or research purposes, to better quantify the adequacy of cleansing attributable to a bowel preparation [6-9]. The validity of a scale indicates how well the scale measures what it is designed to assess, which can be determined via several methods including a comparison with other quality outcomes, e.g., polyp/adenoma detection rate and caecal intubation rate [10]. Validated bowel preparation scales that do not specifically require a fluid score include Boston Bowel Preparation Scale (BBPS), the Chicago Bowel Preparation Scale (CBPS), the Harefield Cleansing Scale (HCS), and the Aronchick bowel preparation [7-10]. Bowel preparation scales evaluating the amount of fluid present or needed for washing or suctioning (e.g., Ottawa Bowel Preparation Scale) are used more for research purposes [11]. The Aronchick Scale is one of the most frequently used validated bowel preparation quality scales in clinical trials and clinical practice {10}. It characterizes the percentage of the total colonic mucosal surface covered by fluid or stool; however, the different colon segments are not scored separately. The scoring is performed before washing or suctioning, hence, a good assessment of the efficacy of a bowel preparation agent is used.

Previous studies have demonstrated that later colonoscopy starting time, a poor compliance with preparation instructions, inpatient status, taking tricyclic antidepressants, male gender, history of diabetes, and prior abdominal surgeries were all independent predictors of an inadequate colon preparation [12,13]. There is a paucity of African literature on inadequate bowel preparation during colonoscopy. To the best of our knowledge, this is the first case-control study on evaluating multiple risk factors for inadequate bowel preparation during colonoscopy in Africa. This study aims to study risk factors of inadequate bowel preparation for colonoscopy in a typical high-fibre diet-consuming Nigerian population in Port Harcourt metropolis and identify those that require an intensified preparation.

## Materials and methods

Study design/setting

A case-control study of patients who underwent colonoscopy in an ambulatory care endoscopy facility with a referral and open-access colonoscopy service, in Port Harcourt, Nigeria from April 2014 to October 2020. Ethical clearance was obtained from Oak Endoscopy Centre Ethics Review Committee (OEM/2020/A006). The data collated included age, gender, literacy level, colonoscopy indication, medical history, preparation agent, the timing of endoscopy procedure, and Aronchick bowel preparation scale Table 1 [11].

**Table 1:** Four-grade modified Aronchick scale for bowel cleansing assessment

Grade	Rating/description
Excellent	Small volume of liquid, >95% of mucosa seen
Good	Clear liquid covering 5-25% of the mucosa, but >90% of mucosa seen
Fair	Semisolid stool could not be suctioned or washed away, but >90% of mucosa seen
Poor	Semisolid stool could not be suctioned or washed away and <90% of mucosa seen

​Selection/matching criteria

Consecutive adult patients who underwent colonoscopy with inadequate bowel preparation irrespective of the indication were retrospectively identified from a total of 546 colonoscopies performed during the study. Excluded in the study were: paediatric patients (age below 18 years); cases of non-compliance with instructions for administration of bowel preparation agents identified from nurses' reports; and incomplete colon study due to obstructing colonic lesion(s). The bowel preparation quality during colonoscopy was categorized using the four-value ordinal modified Aronchick bowel preparation scale; inadequate cases as fair and poor bowel preparation, while adequate (control) for excellent to good bowel preparation [14]. Different bowel preparation agents were used based on availability during different time periods. These were: 4L polyethylene glycol-electrolyte solution (PEG-ELS; Kleanprep, Norgine Ltd., Oxford, UK); two-sachet packs of sodium picosulphate magnesium citrate (Picolax, Ferring Pharmaceuticals, UK); castor oil plus bisacodyl (Dulcolax, Boehringer Ingelheim, Bracknell, UK); eight sachets of PEG 3350 (Movicol, Norgine Ltd., Oxford, UK). Each case of inadequate bowel preparation while using a particular bowel preparation agent was matched (1:1) with a subsequent colon study with adequate bowel preparation (control) for the same agent.

Colonoscopy

Informed consent was obtained from patients before colonoscopy following clear instructions on a minimum of two-day dietary restriction, when to and how to administer agents. A maximum of four hours was kept between the completion of the last preparation agent and the start of colonoscopy for all patients. An information leaflet was given to literate patients with follow-up calls made to all patients on the day preceding the procedure to ensure compliance. Colonoscopies were performed with the patients under conscious sedation by the same surgeon endoscopist (ERO) using a video colonoscope 13925 PKS (Karl Storz SE & Co, Tuttlingen, Germany). A combination of intravenous pentazocine 30 mg and diazepam 5-10 mg was used for sedation in patients in whom there was no contraindication; half the dose of sedative was used in patients over the age of 60 years. Additional sedation was used if required and permissible. Pulse, blood pressure, and oxygen saturation were measured in all patients before, during, and after the procedure. Colonoscopies were performed between 8 am and 2 pm for morning sessions and between 2 pm and 6 pm for afternoon/evening sessions.

Sample size

The sample size (n) was determined using the formula under listed [15].

$n=[(r+1)/r] ((p ̅ )(1- p ̅ ) (Z_&beta;+Z_&alpha;2 )^2)/(p_1-p_2 )^2$

$Z_&beta; =$desired power (1.20 for 90% power)

$Z_&alpha;2 =$desired level of statistical significance (1.96 for 5% significance

$r=$ ratio of control to cases (equal number of cases and control=1)

$p_2=$ proportion exposed to inadequate bowel preparation control; 25% of all colonoscopies [3].

$p_1=$ proportion in exposed cases

For an odds ratio (OR) of 2 or above

$p_2 = 〖ORp〗_(control exposed)/(p_(control exposed) (OR-1)+1) = 2(0.25)/((0.25)(2-1)+1) = 0.5/1.25 = 0.4$

$p ̅ = ((p_1+ p_2 ))/2 p ̅ = ((0.4+0.25))/2 = 0.525$

$n= 2((0.525)(1-0.525) (1.29+1.96)^2)/(0.4-0.25)^2 = 142.5$

Therefore, =286 (143 cases, 143 controls)

Data analysis

The Statistical Package for Social Sciences (SPSS) version 20 (IBM Corp., Armonk, NY) was employed for statistical analysis. Frequencies and proportions were employed for categorical data presentation. The risk factors for inadequate bowel preparation were determined using bivariate and multivariate analysis. The dependent variable was incomplete bowel preparation categorized as Yes/No. The independent variables comprised patients’ age, sex, educational status, co-morbid status of hypertension, and diabetes mellitus. Others were constipation, history of surgery, and timing of endoscopy. The bivariate analysis employed Pearson’s Chi-square with statistical significance set at p<0.25. Variables with p<0.25 from the bivariate analysis were entered into a multivariate analysis model to identify predictors and control for confounders. The multivariate analysis employed a binary logistic regression model, with a significance level of p<0.05.

## Results

Patient characteristics

One hundred and forty-three cases of inadequate bowel preparation during colonoscopy were included in the study with an equal number of controls (cases of adequate bowel preparation). The ages of patients ranged from 24 years to 92 years with patients aged 60 years and above accounting for more than a quarter of cases - 36 (25.2%). There was a male predominance noted with a male/female ratio of 2.7:1. There was a record of post-secondary in just above half of the cases of inadequate bowel preparation - 82 (57.3%) cases. In comparison to 110 (76.9%) of control; a statistically significant difference was noted in the bivariate analysis of education status - p<0.01. The most frequent co-morbidity recorded in patients with inadequate bowel preparation was hypertension - 58 (56.9%). Diabetes mellitus was the next most frequent - 18 (54.5%). There was no record of any patient on psychotropic tricyclic antidepressant drugs or medication for parkinsonism. A bivariate analysis of the timing of colonoscopy from study patients showed no statistical difference between the morning and afternoon procedures (p=0.520; Table 2).

**Table 2:** Bivariate analysis of risk factors associated with inadequate bowel preparation for colonoscopy *Statistically significant p<0.25.

Variables	Number	Inadequate bowel preparation		Chi-square	p-value
		Cases, n (%)	Control, n (%)		
Age					
<40 years	36	16 (44.4)	20 (55.6)	1.864	0.394
40-60 years	187	91 (48.7)	96 (51.3)		
>60 years	63	36 (57.1)	27 (42.9)		
Sex					
Female	74	39 (52.7)	35 (47.3)	0.292	0.589
Male	212	104 (49.1)	108 (50.9)		
Educational level					
Secondary and lower	94	61 (64.9)	33 (35.1)	12.424	0.0001*
Post-secondary	192	82 (42.7)	110 (57.3)		
Hypertension					
Yes	102	58 (56.9)	44 (43.1)	2.987	0.084*
No	184	85 (46.2)	99 (53.8)		
Diabetes mellitus					
Yes	33	18 (54.5)	15 (45.5)	0.308	0.579
No	253	125 (49.4)	128 (50.6)		
Constipation					
Yes	25	16 (64.0)	9 (36.0)	2.148	0.143*
No	261	127 (48.7)	134 (51.3)		
Timing					
Morning	46	25 (54.3)	21 (45.7)	0.414	0.52
Afternoon	240	118 (49.2)	122 (50.8)		

Indications for colonoscopy

Bleeding per rectum was the leading indication for colonoscopy in 122 (42.7%) patients (Table 3).

**Table 3:** Indication for colonoscopy among study patients

Indications for colonoscopy	Frequency	Percentage
Bleeding per rectum	122	42.7
Screening	67	23.4
Abdominal pain/discomfort	43	15.0
Change in bowel habit	33	11.5
Anal protrusion/pain	12	4.2
Abdominal mass	5	1.7
Surveillance	4	1.4
Total	286	100.0

In all, 25 patients presented with constipation from case and control groups, 16 (64%) with inadequate bowel preparation and 9 (36%) controls (p= 0.143).

Bowel preparation agents

**Figure 1:**
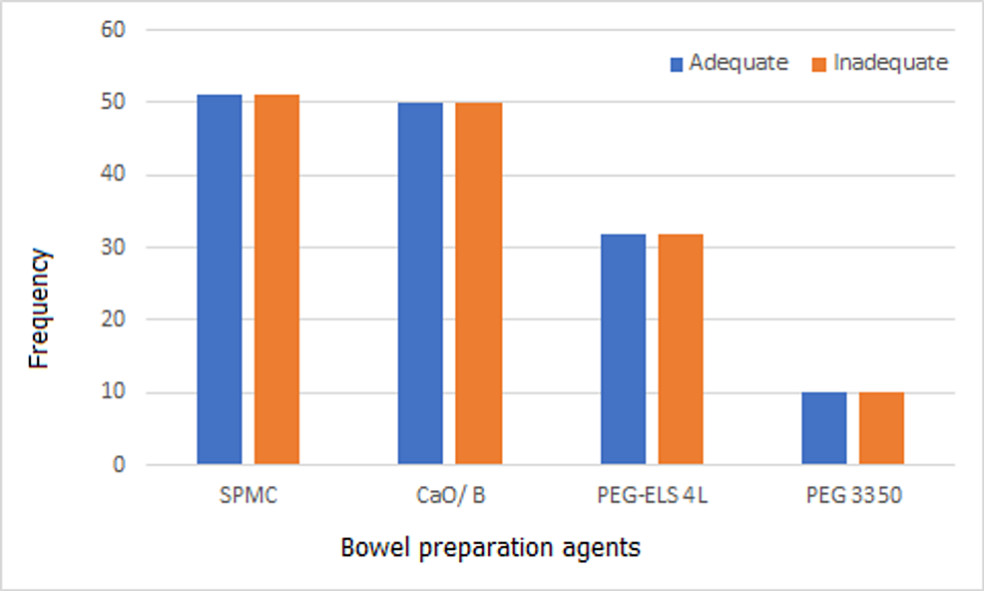
Distribution of bowel preparation agents SPMC: sodium picosulphate magnesium citrate, CaO/B: castor oil and bisacodyl, PEG-ELS 4L: polyethylene glycol electrolyte solution 4L, PEG 3350: polyethylene glycol 3350.

An equal number of cases and control received the given preparation agents according to study protocol (Figure 1).

Multivariate analysis

From logistic regression evaluation of risk factors, secondary level of education and below recorded an OR 2.54, (95% confidence interval CI 1.50-4.30; p=0.001); hypertension - OR 1.64 (95% CI 0.98-2.73; P=0.058); constipation - OR 1.27 (95% CI 0.52-3.10; P=0.598; Table 4).

**Table 4:** Multivariate analysis of risk factors associated with inadequate bowel preparation for colonoscopy ^a^95% confidence interval. ^b^Statistically significant p<0.05.

Variables	Coefficient (B)	Odds ratio	95% CI^a^	p-value
Secondary and below				
Yes	0.93	2.54	1.50–4.30	0.001^b^
No		1	1	
Hypertension				
Yes	0.49	1.64	0.98–2.73	0.058
No		1	1	
Constipation				
Yes	0.24	1.27	0.52–3.10	0.598
No		1	1	

## Discussion

This case-control study conducted on adult patients in Port Harcourt, Nigeria, identified the strongest contributor to inadequate bowel preparation for colonoscopy as literacy level (p<0.01). A lack of comprehension of bowel preparation instructions can contribute to poor compliance especially with split-dose preparations [16]. There is no consensus on the most efficacious way to educate patients about bowel preparation, but multiple different patient education interventions have been demonstrated to improve bowel preparation quality [17]. The US Multi-Society Taskforce on colorectal cancer recommends that all patients receive both oral and written instructions before colonoscopy; however, it does not specify how or when these instructions should be delivered [18]. For this study, verbal instructions were given to all patients, and follow-up telephone calls from the Centre were made on the immediate days preceding colonoscopy in addition to written instructions and literature for literate patients.

The most frequent chronic condition recorded in study patients was hypertension and the most common antihypertensive medication was amlodipine-calcium channel blocker, which has a negative effect on gut motility. Diabetes mellitus was the next most common chronic condition recorded and is associated with reduced colonic and general gastrointestinal transit which may lead to a higher occurrence of inadequate bowel cleaning. From this study, diabetes mellitus was not statistically significant as an independent predictor of inadequate bowel preparation. This study is unlike reports of an odds ratio of 1.8 for inadequate bowel preparation in diabetics and OR 8.6 from Korean patients with a large sample size [12,19]. The reason for the disparity in our study cannot be adequately explained but may be related to compliance rate to clear communication of bowel preparation regime and follow-up calls made on days preceding procedure.

Previous abdominal surgery is reported as a potential risk factor for inadequate bowel preparation, especially gastric and small intestinal surgery [20]. However, this was not demonstrable from study results with less than one-fifth of patients (17.5%) with inadequate bowel preparation having a previous history of abdominal surgery.

Generally, the three commonly used bowel preparation agents are polyethylene glycol (PEG), sodium phosphate, and sodium picosulphate magnesium citrate (SPMC) [21]. The high volume osmotically balanced PEG is popular but due to its taste and large volume, 5% to 15% of the patients do not complete PEG preparations [22]. The use of adjuncts with PEG (e.g., bisacodyl), split dosing and low volume preparations improve patient acceptability with effective outcomes [6]. Sodium phosphate preparations are hyperosmotic and effective but there are concerns regarding the safety of this agent [23]. SPMC is a low-volume, dual-action (osmotic and stimulant) laxative easy to administer, and well-tolerated bowel preparation agent [24]. In limited-resource settings, there is a report of the use of castor oil and bisacodyl; however, with a need for copious washing and suctioning [25]. This regime was used in interval periods when the preferred bowel preparation agents were exhausted with non-availability of local supply or due to delays from importation challenges.

The age distribution of patients with inadequate bowel preparation was observed to have recorded 63 (22.0%) aged greater than 60 years. It is recognized that elderly patients have decreased colon transit, increased comorbidity, and polypharmacy; all of which are known risk factors for poor colon cleansing [26-28]. A similar study of a large African American population confirmed reported risk factors for inadequate preparations, including age, sex, and afternoon colonoscopy [29]. However, in the bivariate analysis of data from this study, there was no statistical difference between the mean age of patients with inadequate bowel preparation and controls (55.0 years vs 53.1, p=0.187).

A limitation to this study is the retrospective study design. There were no data available on in-hospital status which may negatively influence the effectiveness of bowel preparation agents. Also, this is a single centre study, a generalization of finding across Nigeria and similar African populations may be uncertain though our findings are consistent with prior reports in world literature.

## Conclusions

The educational status of patients was the strongest contributor to inadequate bowel preparation for colonoscopy in our Nigerian population. There is a need for effective patient education especially for patients with a low literacy level beyond verbal instructions on bowel preparation prior to colonoscopy. The dual communication of bowel preparation instruction to the patient and a literate, responsible relative to include written instructions and literature is advisable.

In all, comprehension of bowel preparation instruction and compliance are cardinal to adequate bowel preparation irrespective of the presence of other mitigating factors.
